# Synergistic Antibacterial Effects of Polyphenolic Compounds from Olive Mill Wastewater

**DOI:** 10.1155/2011/431021

**Published:** 2011-05-02

**Authors:** Ahmed Tafesh, Naim Najami, Jeries Jadoun, Fares Halahlih, Herbert Riepl, Hassan Azaizeh

**Affiliations:** ^1^The Institute of Applied Research (Affiliated with University of Haifa), The Galilee Society, P.O. Box 437, Shefa-Amr 20200, Israel; ^2^Department of Biology, The Academic Arab College of Education, Haifa 33145, Israel; ^3^Institute of Resource and Energy Technology, Technical University of Munich, Schulgasse 16, 94315 Straubing, Germany

## Abstract

Polyphenols or phenolic compounds are groups of secondary metabolites widely distributed in plants and found in olive mill wastewater (OMW). Phenolic compounds as well as OMW extracts were evaluated *in vitro* for their antimicrobial activity against Gram-positive (*Streptococcus pyogenes* and *Staphylococcus aureus*) and Gram-negative bacteria (*Escherichia coli* and *Klebsiella pneumoniae*). Most of the tested phenols were not effective against the four bacterial strains when tested as single compounds at concentrations of up to 1000 *μ*g mL^−1^. Hydroxytyrosol at 400 *μ*g mL^−1^ caused complete growth inhibition of the four strains. Gallic acid was effective at 200, and 400 *μ*g mL^−1^ against *S. aureus*, and *S. pyogenes*, respectively, but not against the gram negative bacteria. An OMW fraction called AntiSolvent was obtained after the addition of ethanol to the crude OMW. HPLC analysis of AntiSolvent fraction revealed that this fraction contains mainly hydroxytyrosol (10.3%), verbascoside (7.4%), and tyrosol (2.6%). The combinations of AntiSolvent/gallic acid were tested using the low minimal inhibitory concentrations which revealed that 50/100–100/100 *μ*g mL^−1^ caused complete growth inhibition of the four strains. These results suggest that OMW specific fractions augmented with natural phenolic ingredients may be utilized as a source of bioactive compounds to control pathogenic bacteria.

## 1. Introduction

The process of olive oil production is accompanied by generation of a considerable amount of olive mill wastewater (OMW). Up to 30 million m^3^ of OMW is produced annually in the Middle Eastern countries during the olive oil processing. The OMW is rich with organic compounds (mainly phenols) which creates a number of acute environmental and ecological problems [1, 2]. So far, there is no accepted treatment method for all the wastes generated during olive oil production [3]. However, several approaches to treat the OMW have been suggested including anaerobic biodegradation [4, 5], detoxification by fungi [6], ozonation [7], as well as other new bioremediation and biovalorisation strategies [3]. 

The phenolic fraction of olive oil comprises only 2% of the total phenolic content of the olive fruits, with the remaining 98% being lost in olive mill waste (OMW) [8]. Thus, OMW is also potentially a rich source of a diverse range of phenols with a wide array of biological activities. The OMW itself is phytotoxic; however it possesses antimicrobial activity due to the phenolic compounds present in the waste [9, 10]. A number of studies have shown that these compounds are effective as antibacterial, antiviral, and antifungal compounds [11–14]. Research into finding new uses for by-products of olive oil production is of great interest not only to the economy but also to the environment, particularly in areas where olives are grown and OMW is wasted [1, 15]. 

Phenols and polyphenols are diverse group of compounds which widely occur in a variety of plants including olives and are used in defensive functions in many plant species [14, 16] where some of which enter into the food chain and some used as antimicrobial products [16–21]. They also represent natural anti-inflammatory agents [22] used to replace the synthetic drugs which cause side effects [23, 24]. Research studies on bioactive compounds showed that single phenolic compounds or their combination resulted in growth inhibition of different bacterial strains [25, 26]. The minimal inhibitory concentration (MIC) of both* p*-coumaric and caffeic acids against *Xylella fastidiosa* strains (causes Pierce's disease in grapes) were 800 *μ*M and 200 *μ*M, respectively [27]. Compounds found in OMW that exhibited antibacterial activity were hydroxytyrosol [28], oleuropein and hydroxytyrosol [29], 4-hydroxybenzoic acid, vanillic acid, and *p-*coumaric acid [30]. Olive polyphenols such as hydroxytyrosol have been found to act *in vitro* against both Gram-positive and Gram-negative bacteria responsible for respiratory and intestinal tract infections [31]. In a recent study, the addition of OMW to soil exerted significant disease suppressiveness against the soil-borne diseases caused by *Rhizoctonia solani* and* Fusarium solani* [32]. 

A large number of research papers have been published dealing with the chemical composition of olives and olive oil; however, only a few studies have focused on isolating and identifying compounds from the OMW [33]. The isolation of these bioactive metabolites, especially tyrosol and hydroxytyrosol, aromatic acids, and conjugated aromatic acids from the OMW, is of great interest particularly because of their antioxidant and antimicrobial properties [29, 30, 34, 35]. In most of the above-mentioned studies, extracts from OMW or synthetic compounds were tested against different microorganisms and some were found effective and others with less or no activity. 

The aims of the current research were (a) to develop a simple and cost-efficient OMW extraction method yielding a highly active antimicrobial phenolic fraction effective against important human pathogenic bacteria, (b) to define the active constituents (and/or phenolic compounds) of such fraction (using pure compounds), and (c) to investigate the synergistic effects of known bioactive compounds and OMW fractions against human pathogenic bacteria.

## 2. Materials and Methods

### 2.1. Standards and Phenolic Compounds

Phenolic and other standards used without further purification were ascorbic acid, tyrosol, protocatechuic acid, vanillic acid, caffeic acid, gallic acid, ferulic acid, and *p*-coumaric acid from Sigma-Aldrich Ltd, Israel, hydroxytyrosol, from TCI AMERICA, 3,4-dihydroxyphenylacetic acid from ACROS chemicals, and verbascoside from Apin Chemicals Ltd, UK.

### 2.2. Bacterial Strains

The tested bacterial strains included the Gram-positive reference strains *Streptococcus pyogenes* (ATCC no. 19675) and *Staphylococcus aureus* (ATCC no. 25923) and the Gram-negative reference strains *Escherichia coli* (ATCC no. 25922) and *Klebsiella pneumoniae* (ATCC no. 700603). Bacterial strains were maintained on tryptic soy broth (TSB) containing 20% glycerol and stored at −80°C until use. Subcultures were freshly prepared before use by inoculation of a loop of stored culture into 5 mL TSB and incubation overnight at 37°C. The turbidity of the culture was adjusted with sterile saline solution to match 0.5 McFarland standards (Center for Disease Control and Prevention Antimicrobial Susceptibility testing (agar Disk Diffusion Method) (http://www.cdc.gov/ncidod/DBMD diseaseinfo/cholera/ch9.pdf).

### 2.3. Olive Mill Wastewater (OMW)

The OMW was generated by the olive oil extraction using the three-phase known process. OMW for this study was obtained from a nearby olive mill press (Iksal, Galilee region, Israel). The OMW was treated with 20% ethanol (v : v) and stored at 4°C until use. The total phenol (TP), COD, BOD, and pH values, of the collected OMW samples were determined according to the “Standard Methods for the Examination of Water and Wastewater, 20th Edition 1998.” The TP in OMW was determined according to the Folin-Ciocalteu method [36].

### 2.4. Preparation of the AntiSolvent Fraction

**Figure 1:**
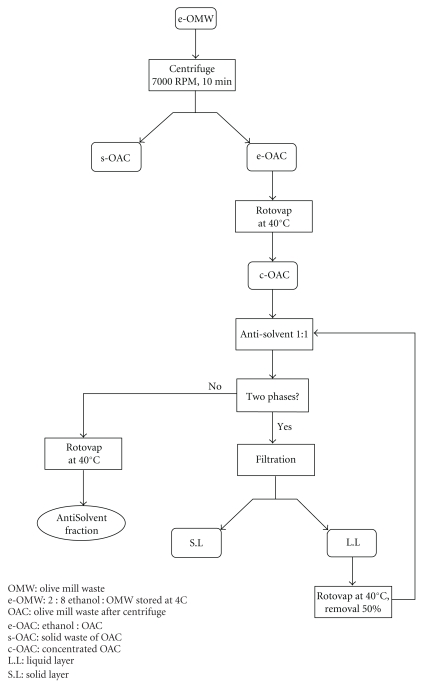
Extraction process of the AntiSolvent fraction from OMW.

The AntiSolvent was prepared in a very unique way in order to extract polyphenols from OMW. The AntiSolvent fraction was obtained by the addition of at least one polar organic solvent (acetone or ethanol) to the aqueous mixture. The polar solvent caused a precipitation and therefore forces out an organic fiber fraction from the solution which was identified mainly as cellulosic mixture (no phenolic chromophore was identified using the HPLC). The AntiSolvent used throughout these experiments was prepared as follows. One liter of OMW stored at 4°C with 20% ethanol was centrifuged (7000 rpm for 10 min) and then subjected to filtration using Wattman filter paper (Figure 1). The resulting e-OMW was filtered through two layers of gauze to get an organic fraction mixed with 20% ethanol (e-OAC). The e-OAC was concentrated under high vacuum using a rotory evaporator until it reached a volume of 250 mL (c-OAC). Additional 250 mL of 95% ethanol was added to the c-OAC to give two phases (a solid precipitate and a liquid layer). The solid phase was removed from the mixture by filtration, and the liquid phase was evaporated at 40°C using rotory evaporator to produce approximately 250 mL volume. The process of evaporation and addition of 95% ethanol was repeated until no more solid (cellulosic mixture) precipitated from the OAC fraction remained. The liquid phase which mainly contains polyphenolic mixture was evaporated under high vacuum to produce 10.0 g of a dark-brown paste which was called AntiSolvent (Figure 1). The AntiSolvent fraction was stored at 4°C and thereafter used to test its antimicrobial potential in a biological test, to identify the compounds, and to quantify the phenolic content of each compound; the extract was redissolved in methanol and analysed using HPLC-PAD techniques.

### 2.5. Phenolic Compounds Analysis

The presence and amount of the phenolic compounds in the AntiSolvent extract were studied using reversed-phase HPLC analysis with a binary gradient elution. The analysis was performed by reversed-phase HPLC using a Thermo Scientific Finnigan Surveyor system equipped with a PDA plus detector (220–340 nm). The chromatographic separation was achieved on a SYNERGI 4U POLAR-RP 80A 250 × 4.60 mm phenomenex. Its temperature was maintained at 30°C. The mobile phase was 0.1% acetic acid in water (A) versus 0.1% acetic acid in methanol (B) for a total running time of 40 min. The specific elution conditions were 0–5 min, 20% B; 5–10 min, 20–70% B; 10–21 min, 70–80% B; 21–30 min, 80% B; 30–32 min, 80–20% B; 32–40 min, 20% B. The flow rate was 1.0 mL/min, and the injection volume was 20 *μ*L. The main phenolic compounds in the extract were identified and quantified by comparison with relative retention times and UV spectra of pure standards (Sigma-Aldrich Ltd, Israel; TCI AMERICA; Apin Chemicals, Ltd,UK; ACROS chemicals).

### 2.6. Antibacterial Activity

The inoculums were prepared by lifting 3–5 identical colonies from each agar plate with a sterile loop and transferred into a tube containing 5 mL of TSB and incubated overnight at 37°C. The turbidity of each bacterial suspension was adjusted to reach an optical comparison to that of a 0.5 McFarland standard, resulting in a suspension containing approximately 1 − 2 × 10^8^ cfu mL^−1^. Each fraction/component or combination of compounds was examined for antibacterial activity in triplicate wells using 96-well plates, and the experiments were repeated at least twice. The plates were incubated at 37°C for 18 h. Subsequently, the plates were examined visually for bacterial growth inhibition. In each treatment, the inhibition was considered positive when there was no microbial growth in all the 3 wells of the triplicate. The antimicrobial activity of the different compounds and mixtures was tested against Gram-positive (*S. pyogenes* and *S. aureus*) and Gram-negative bacteria (*E. coli* and *K. pneumoniae*) in order to determine the MIC for the different combinations. The MIC was determined as the lowest combination of two compounds caused complete growth inhibition in the triplicate wells of each treatment.

## 3. Results

### 3.1. Isolation of AntiSolvent Fraction

**Figure 2:**
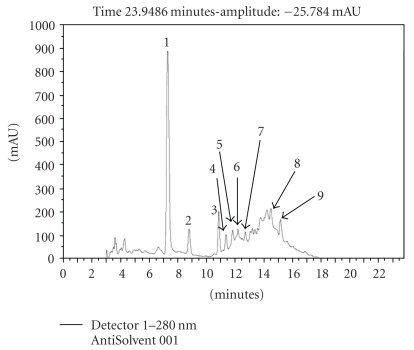
Chromatographic profile of the AntiSolvent extract of OMW obtained by HPLC-PAD detected at the relative maxima of absorbance of polyphenols (280 nm). Key to peak identities: (1) hydroxytyrosol; (2) 3,4-dihydroxyphenylacetic acid; (3) tyrosol; (4) protocatechuic acid; (5) verbascoside; (6) vanillic acid; (7) caffeic acid; (8) ferulic acid; (9) *p*-coumaric acid.

**Figure 3:**
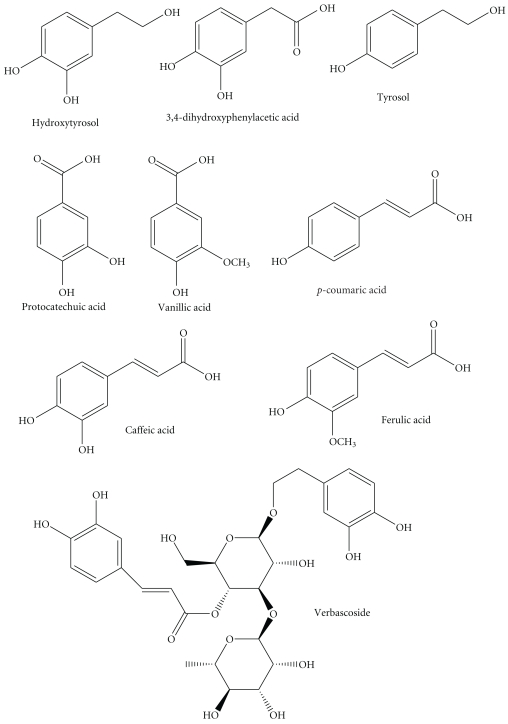
Structures of bioactive phenolics in the AntiSolvent paste.

The OMW used in our experiments obtained from our Galilee region and containing total phenols 6.6, COD 170.2, BOD 27.5 g l^−1^, and the pH was 5.0. The AntiSolvent fraction was isolated without tedious extraction method for ease of isolation. The dark AntiSolvent liquid evaporated to give 10.0 grams of brown/black thick paste from 1 liter of OMW. The content of the paste was identified using HPLC method and constituted of hydroxytyrosol, 3, 4-dihydroxyphenylacetic acid, tyrosol, protocatechuic acid, verbascoside, vanillic acid, caffeic acid, ferulic acid, and *p*-coumaric acid in addition to other unidentified peaks (Figures 2 and 3). The amounts of these compounds, calculated based on 1000 ppm AntiSolvent extract of OMW, and the main constitutes were as follows: hydroxytyrosol (102.9 ppm), verbascoside (73.9 ppm), tyrosol (26.1 ppm), ferulic acid (15.7 ppm), and *p*-coumaric acid (14.3 ppm) (Table 1). 

### 3.2. Antimicrobial Activity

#### 3.2.1. Antimicrobial Activity of Single Compounds

The antimicrobial activity of the AntiSolvent fraction and different single phenolic compounds obtained from our OMW in addition to some other compounds was tested as well. The other compounds were selected because there are some reports that these compounds are used as antimicrobial bioactives. The AntiSolvent fraction caused inhibition to *E. coli* and *S. pyogenes *at 1000 *μ*g mL^−1^ as was visually observed (Table 2). Hydroxytyrosol at 400 *μ*g mL^−1^ caused growth inhibition to the four bacterial isolates. Tyrosol at 600 *μ*g mL^−1^ caused growth inhibition to 3 isolates, except *S. aureus. *Ascorbic acid inhibited the growth of *S. pyogenes* only at 400 *μ*g mL^−1^. Gallic acid at 200 and 400 *μ*g mL^−1^ inhibited the growth of *S. aureus* and *S. pyogenes *strains, respectively. No growth inhibition was observed for the Gram-negative bacteria (*E. coli* and *K. pneumoniae*) when gallic acid was supplemented up to 1000 *μ*g mL^−1^ (Table 2). Caffeic, ferulic, *p*-coumaric, cinnamic, vanillic, protocatechuic, and syringic acid supplemented separately up to 1000 *μ*g/mL resulted in no growth inhibition of the four bacterial strains (Table 2).

#### 3.2.2. Synergistic Effects of Different Combinations as Antimicrobial Compounds

The AntiSolvent fraction alone caused inhibition to *E. coli* and *S. pyogenes *at 1000 *μ*g mL^−1^ (Table 2). Since we were able to characterize many constitutes of the AntiSolvent fraction, we decided to test which compounds are the most active. Is the antimicrobial activity related to single compounds or more or all together? Can the antimicrobial effect be augmented by enrichment with other known olive or OMW compounds? Therefore we decided to move toward testing synergistic effects because the AntiSolvent fraction did not contain all the compounds we tested in the first stage.  Table 3 summarizes the synergistic antimicrobial and the MIC of the different mixtures of AntiSolvent with hydroxytyrosol, or gallic acid in addition to the combinations of hydroxytyrosol, gallic, and ascorbic acid. The results show very clearly the synergistic effect of these combinations. The combination of AntiSolvent/hydroxytyrosol (400/200 *μ*g mL^−1^) resulted in complete inhibition of the four strains. Also, it would require the combination of gallic acid/hydroxytyrosol (100/200 *μ*g mL^−1^) to completely inhibit the growth of the same four bacterial isolates. It is interesting to note that ascorbic acid/hydroxytyrosol showed synergistic activity against the four isolates and resulted in complete inhibition to *S. pyogenes *at the combination 100/50 *μ*g mL^−1^. The combination of AntiSolvent/hydroxytyrosol in MIC of 50/50 *μ*g mL^−1^ resulted in complete inhibition of the isolate* S. pyogenes* (Table 3). Other combinations were tested as well but the results were not encouraging (data not shown).

## 4. Discussion

The increasing occurrence, particularly in hospitals, of pathogenic resistant bacteria especially *S. aureus *to a wide range of antimicrobial agents, including all kinds of *β*-lactams, has made therapy more difficult. The increasing resistance to antibiotic represents the main factor justifying the need to find and/or develop new antimicrobial agents. Thus, many studies have been focused on antimicrobial agents and on the antimicrobial properties of plant-derived active principles [10, 16, 26, 37]. Although strategies have been proposed in an attempt to control the spread of pathogenic bacteria, the search for new ways to treat infections stimulates the investigation for natural compounds as an alternative treatment of these infections. In our search for antimicrobial ingredients from OMW we choose to use fractions and synergy of at least 2 compounds for several reasons. First, in a general way, the antimicrobial capacity of phenolic compounds is well known [38, 39]. In addition, extracts (fractions) may be more beneficial than isolated constituents, since a bioactive individual component can change its properties in the presence of other compounds present in the extracts [40]. According to Liu [41], additive and synergistic effects of phytochemicals in fruits and vegetables are responsible for their potent bioactive properties, and the benefit of a diet rich in fruits and vegetables is attributed to the complex mixture of phytochemicals present in whole foods. This explains why no single antimicrobial can replace the combination of natural phytochemicals to achieve the health benefits. Some researchers have also demonstrated that biocompounds present in olive products, such as oleuropein [42, 43] and hydroxytyrosol [42] and aliphatic aldehydes [44], inhibit or delay the rate of growth of a range of bacteria and microfungi, so that they might be used as alternative food additives or in integrating pest management programs [45]. Therefore, in the current research we focused on extraction of unique fractions from the OMW and test combinations of compounds since single compounds or fractions demonstrated low inhibition effects, and in addition OMW fraction did not contain some important phenolic compounds such as gallic acid. 

The growth inhibition of the different bacterial strains was tested using the broth dilution method which showed synergistic activity of the AntiSolvent fraction obtained from OMW in combination with hydroxytyrosol or with gallic acid (Table 3). Also the combination hydroxytyrosol/gallic acid resulted in positive synergistic effects against the four bacterial isolates. However, when many phenolic compounds were tested as single compounds at up to 1000 *μ*g mL^−1^ no growth inhibition was observed (Table 2). Mixtures of phenolic compounds were detected in our AntiSolvent fraction obtained from the OMW which indicates that the natural combination of these compounds is better than using single compound as antimicrobial compound. The OMW is rich with hydroxytyrosol (102.9 ppm), verbascoside (73.9 ppm), and tyrosol (26.1 ppm), but no gallic acid was detected (Table 1). The bioactivity of the single phenolic component (in most cases) used in the current study against the Gram-positive (*S. pyogenes* and *S. aureus*) and the Gram-negative bacteria (*E. coli* and *K. pneumoniae*) was found to be very low and required high concentration exceeding 1000 *μ*g mL^−1^ per component to inhibit the growth of the four isolates (data not shown) except for hydroxytyrosol. However, an enriched AntiSolvent with combinations of specific phenolic compounds completely inhibited all four bacterial strains at different combinations with low concentration combinations of 50/50–200/400 *μ*g mL^−1^. The MIC of the phenolic compounds extracted from olives (the phenolic amounts found in table olives ranged from 0.9 to 5 g/kg) was established against bacterial isolates responsible for human intestinal and respiratory tract infections such as *Bacillus cereus* (10,000 *μ*g mL^−1^), *B. Subtilis* (100,000 *μ*g mL^−1^), *S. aureus* (50,000 *μ*g mL^−1^), *Pseudomonas aeruginosa* (100,000 *μ*g mL^−1^), *E. Coli* (75,000 *μ*g mL^−1^), and *Klebsiella pneumoniae* (50,000 *μ*g mL^−1^) [38]. These are high MIC values compared to our results where combinations of 50/50–200/400 *μ*g mL^−1^ of AntiSolvent with hydroxytyrosol or with gallic acid caused complete inhibition to the four microbial strains. 

The AntiSolvent fraction at 1000 *μ*g mL^−1^ alone completely inhibited the growth of two isolates only, *E. coli* and *S. pyogenes*. Also hydroxytyrosol which is known as antimicrobial compound [42] required at least 400 *μ*g mL^−1^ to cause full growth inhibition of the four strains. However, the combination of various phenolic compounds was effective against the four different bacterial strains because of the synegistic effect obtained using various components, that is, AntiSolvent/hydroxytyrosol, AntiSolvent/gallic, and gallic/hydroxytyrosol (Table 3). The HPLC analysis of the AntiSolvent obtained from the OMW revealed that this fraction contains mainly hydroxytyrosol (102.9 ppm), verbascoside (73.9 ppm), and tyrosol (26.1 ppm) (Table 1). An ethyl acetate extract of a Tunisian OMW showed high hydroxytyrosol and tyrosol concentrations of 690 and 98 mg g^−1^ dry weight extract, respectively [46]. The analysis of phenolic compounds of different table olives from Portugal was performed using reversed-phase HPLC/DAD, where seven compounds were identified and quantified: hydroxytyrosol, tyrosol, 5-O-caffeoilquinic acid, verbascoside, luteolin 7-O-glucoside, rutin, and luteolin [38]. In their study hydroxytyrosol, tyrosol, and luteolin were the prevailing phenols in all samples. Using different analysis techniques we have shown that hydroxytyrosol, verbascoside, and tyrosol were the prevailing phenols in our OMW AntiSolvent fraction (Table 1, Figure 2). 

The hydroxytyrosol alone was effective against all the 4 strains at 400 *μ*g (Table 2). Enrichment of the AntiSolvent with pure hydroxytyrosol reduced the amount of both the AntiSolvent and the hydroxytyrosol. Also gallic acid was active only when combined with hydroxytyrosol or with AntiSolvent. Taken together, these findings suggest that hydroxytyrosol is the main bioactive compound in the AntiSolvent fraction and it is an important factor in growth inhibition (Table 3). In another study, more than 18 compounds including hydroxytyrosol glucoside, hydroxytyrosol, tyrosol, caffeic acid, verbascoside, luteolin glycoside, rutin, and verbascoside isomer were detected in two fractions called FOE and MOE [47]. These two fractions showed broad spectrum antibacterial activity against* S. aureus, B. subtilis, E. coli*,* and P. aeruginosa*, whereas individual phenols (hydroxytyrosol, luteolin, and oleuropein) showed more limited activity [47]. 

An interesting result was obtained by the combination of hydroxytyrosol/ascorbic acid (vitamin C) where 200/200 *μ*g mL^−1^ and even less caused full growth inhibition of the four bacterial isolates (Table 3). The explanation for the high antibacterial effect of hydroxytyrosol/ascorbic acid could be due to some additive effects of both compounds. The combination of other components was less effective (data not shown). 

Recovery of phenols from OMW is a difficult analytical task for several reasons. Phenols are reactive chemical species, vulnerable to oxidation, conjugation, hydrolysis, polymerization, and complexation [48]. This is compounded by direct contact with enzymes and their substrates as the cells are no longer intact. OMW is a complex matrix that offers a reaction medium (water), catalysts (enzymes, organic acids, and metals), and substrates (proteins, polysaccharides, metals, small-molecular-weight reactive compounds, and phenols themselves), all contained under an umbrella of oxygen (air). Olive comprises a vast range of phenolic compounds with different structures and different physicochemical properties (solubility and partitioning) that makes any attempt to optimize the extraction a difficult task [48]. In many instances, the nature of the sample and details of sample handling prior to extraction are omitted. In those cases where details are provided, there is great diversity. For instance, Visioli and Galli [18] used fresh OMW derived from bench-top milling of frozen olives, whereas Capasso et al. [28] used fresh commercial OMW. The immediate analysis of the fresh sample [49] is always the ideal situation, due to possible changes in the chemical composition during sample manipulation. Unfortunately, this is rarely achievable, and sample transfer to the laboratory, preservation, and storage may be unavoidable and affect the results. In our study the OMW was treated with 20% ethanol and stored at 4°C until use; therefore, our fraction might be exposed to less chemical changes.

The most important conclusion drawn from our study is that simple, efficient, and cost-effective extraction of OMW yielding highly active antimicrobial extract can be done and the extract can be further augmented with additional natural compounds to achieve higher activity. These finding may lead to more attention to natural compounds as an alternative treatment of infectious diseases [35, 38]. Such combination mixtures which were found to be efficacious against the four different pathogens (Table 3) will be evaluated for their use as formulations of drugs for prevention or treatment of bacterial infections.

## Figures and Tables

**Table 1:** Retention time and calculated concentrations of the different compounds isolated and identified from 1000 ppm AntiSolvent extract of OMW obtained by HPLC-PAD. The results are the mean of 3 replicates and based on the calculation of the area of injected standards. The concentration was calculated as area/slope of each peak and presented as means ± SD.

Compound	Retention time (min)	Area	Slope	*R* ^2^	Concentration (ppm)
Hydroxytyrosol	7.105	12838384	124745	0.9857	102.9 ± 1.1
3,4-dihydroxyphenylacetic acid	8.865	2103719	203297	0.9959	10.3 ± 0.4
Tyrosol	10.775	2274965	87195	0.9660	26.1 ± 0.2
Protocatechuic acid	11.315	1620535	118665	0.9877	13.7 ± 0.1
Verbascoside	11.793	2295010	31060	0.9854	73.9 ± 0.6
Vanillic acid	12.155	3349562	274065	0.9901	12.2 ± 0.3
Caffeic acid	12.750	2029414	277494	0.9922	7.3 ± 0.3
Ferulic acid	14.723	4003895	254636	0.9495	15.7 ± 0.3
*p*-coumaric acid	15.463	3448623	241817	0.9990	14.3 ± 0.3

**Table 2:** Minimal inhibitory concentration (MIC) of phenolic compounds against the Gram-positive (*S. pyogenes and S. aureus*) and Gram-negative bacteria (*E. coli and K. pneumoniae*). Each well contains ~10^5^ cfu. The results were obtained after incubation at 37°C for 24 h. NE represents no growth inhibition.

	Strain			
	*E. coli*	*S. pyo*g*enes *	*K. Pneumoniae*	*S. aureus*
Component (*μ*g/mL)				
AntiSolvent	1000	1000	NE	NE
Hydroxytyrosol	400	400	400	400
Tyrosol	600	600	600	NE
Gallic acid	NE	400	NE	200
Ascorbic acid	NE	1000	NE	NE
Caffeic acid	NE	NE	NE	NE
Ferulic acid	NE	NE	NE	NE
Coumaric acid	NE	NE	NE	NE
Cinnamic acid	NE	NE	NE	NE
Vanillic acid	NE	NE	NE	NE
Syringic acid	NE	NE	NE	NE
Protocatechuic acid	NE	NE	NE	NE

**Table 3:** MIC of the different combinations used against *S. pyogenes, S. aureus, E. coli*, and *K. pneumoniae*. Each well contains ~10^5^ cfu. The results were obtained after incubation at 37°C for 24 h.

	Strain			
	*E. coli*	*S. pyogenes*	*K. pneumoniae*	*S. aureus*
Synergy (*μ*g/mL)				
AntiSolvent	400	50	200	200
Hydroxytyrosol	200	50	200	100

AntiSolvent	50	50	100	50
Gallic acid	100	100	100	100

Gallic acid	100	50	100	100
Hydroxytyrosol	200	100	50	50

Hydroxytyrosol	200	50	100	100
Ascorbic acid	100	100	200	100
